# Large Language Models in Medicine: Applications, Challenges, and Future Directions

**DOI:** 10.7150/ijms.111780

**Published:** 2025-05-31

**Authors:** Erlan Yu, Xuehong Chu, Wanwan Zhang, Xiangbin Meng, Yaodong Yang, Xunming Ji, Chuanjie Wu

**Affiliations:** 1 Department of Neurology, Xuanwu Hospital, Capital Medical University, Beijing, China.; 2 Pengcheng Laboratory, Shenzhen 518055, P. R. China.; 3 Institute for AI, Peking University.

**Keywords:** Large language models, Medical applications, Natural language processing, Artificial Intelligence

## Abstract

In recent years, large language models (LLMs) represented by GPT-4 have developed rapidly and performed well in various natural language processing tasks, showing great potential and transformative impact. The medical field, due to its vast data information as well as complex diagnostic and treatment processes, is undoubtedly one of the most promising areas for the application of LLMs. At present, LLMs has been gradually implemented in clinical practice, medical research, and medical education. However, in practical applications, medical LLMs still face numerous challenges, including the phenomenon of hallucination, interpretability, and ethical concerns. Therefore, in-depth exploration is still needed in areas of standardized evaluation frameworks, multimodal LLMs, and multidisciplinary collaboration in the future, so as to realize the widespread application of medical LLMs and promote the development and transformation in the field of global healthcare. This review offers a comprehensive overview of applications, challenges, and future directions of LLMs in medicine, providing new insights for the sustained development of medical LLMs.

## Introduction

Large language models are deep learning models based on the Transformer architecture, which leverages the self-attention mechanism. They are not only capable of generating natural language text, but also capable of deeply understanding the meaning of the text and processing various natural language tasks, such as text summarization, and question answering 1. In 2022, OpenAI released ChatGPT, which quickly attracted attention and heated discussion of all walks of life 2. Since then, LLMs exemplified by ChatGPT have been widely used in various fields and have achieved significant breakthroughs, such as OpenAI o1 in Mathematics and Programming.

Currently, the field of medicine is undergoing rapid development, and there is an urgent need to introduce new tools or explore innovative approaches to solve existing problems. LLMs have paid much attention to clinical experts in recent years due to their powerful natural language processing (NLP) capabilities. It has become a research hotspot in medicine, bringing unprecedented development opportunities to the field. In clinical practice, LLMs can assist doctors in optimizing clinical decisions by analyzing patient information 3. In medical research, LLMs can assist in paper writing, mining and analyzing data, thus improving research efficiency 4. In medical education, LLMs can simulate real patients and act as virtual teaching assistants, providing personalized learning programs 5. Despite the great potential of LLMs in medicine, they still face numerous challenges, such as hallucinations, its black-box nature, the lack of evaluation benchmarks and high-quality data, energy consumption and ethical concerns, which severely limit their practical application 6, 7. Therefore, it is crucial to summarize and analyze the current research status and development trend of LLMs in medicine.

In this review, we provide a systematic and comprehensive overview of the applications and challenges of LLMs in medicine, along with specific recommendations for their future development, aiming to offer valuable references to clinicians and researchers.

## Development of large language models

### Progress and innovations in LLMs

**Figure 1:**
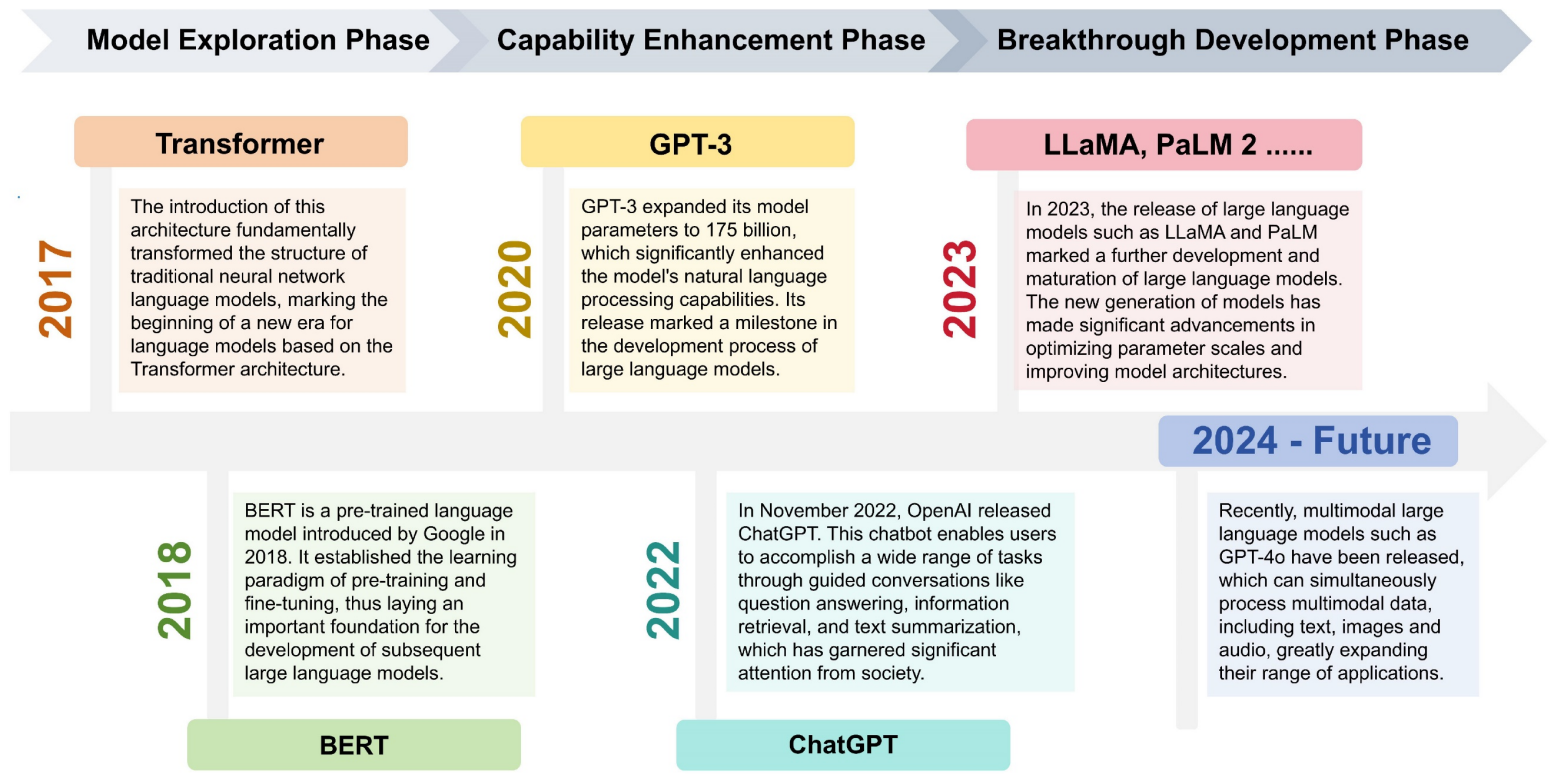
Development timeline of LLMs.

LLMs refer to language models with over hundreds of billions of parameters, which are trained on vast amounts of text data 8. In 2018, Google released BERT, a pre-trained language model that pioneered the learning paradigm of “pre-training and fine-tuning”, improving performance on NLP tasks to a large extent 9. In the same year, OpenAI also released the generative pre-training model GPT 10. Since then, pre-trained language models have begun to come into the public eye. In 2020, the release of GPT-3 with a parameter scale of 175 billion officially opened the era of LLMs 1. In November 2022, OpenAI released ChatGPT, which was an important milestone in the development process of LLMs 2. Subsequently, LLMs entered a phase of rapid development. Meta, Google, Anthropic and other companies released multiple LLMs like LLaMA 11, PaLM 2 12, Gemini, and Claude which performed excellently in NLP tasks (Figure 1).

In recent years, a growing number of medical LLMs have emerged, such as Med-PaLM, which is based on PaLM. The study showed that Med-PaLM was the first LLM to achieve a passing score on the United States Medical Licensing Examination (USMLE). It was not only comparable with clinicians in medical knowledge retrieval, but also demonstrated significant advantages in answering patients' medical questions 13. Additionally, Med-PaLM 2 was the first LLM to reach the level of human experts in answering USMLE-style questions. It could correctly answer multiple-choice and open-ended questions with an accuracy of up to 86.5% 14.

### The principles of LLMs

Currently, LLMs typically undergo two stages: first, acquiring NLP capabilities through pre-training, and then further optimizing the model for specific domains through post-training. Pre-training is the initial stage of language model learning, usually adopting the framework based on the Transformer model. The models learn from large-scale unlabeled text data in an unsupervised manner, capturing the linguistic patterns, structures, and grammar in the text corpus. This process enables models to understand the contextual information and semantic relationships in the text, while equipping them with rich vocabulary knowledge 9, 15. Post-training refers to further adjusting and optimizing the model through methods like fine-tuning and alignment to improve its performance on specific tasks. Fine-tuning is the process of further training LLMs using task-specific datasets, which is an effective parameter calibration technique. The FLAN model released by Google first introduced the paradigm for instruction fine-tuning, enabling the model to better respond to human instructions and thereby generating accurate feedback 16.

In addition, prompt engineering is employed in practical applications to efficiently invoke the powerful capabilities of LLMs. It refers to the design, optimization, and implementation of prompts and instructions, which helps users apply LLMs to various scenarios and research fields. As a matter of fact, it is a practice of effectively interacting with artificial intelligence (AI) systems to optimize their performance 17. In the future, prompt engineering is expected to become an important bridge between users and LLMs.

### Comparative Overview of Leading LLMs

In recent years, several representative LLMs have emerged, each demonstrating unique advantages in architectural design and practical deployment. ChatGPT, developed by OpenAI, has shown outstanding performance in NLP, with strong capabilities in understanding complex language structures and semantics, generating logically coherent and content-rich responses 2. In the medical domain, ChatGPT has demonstrated potential for clinical decision support. Studies have shown that physicians assisted by GPT-4 perform significantly better in complex case management than those relying on traditional methods 18. The release of GPT-4o in 2024 further enhances the model's response speed and operational efficiency, making it suitable for a wide range of task scenarios 19. The newly launched OpenAI o1 integrates reinforcement learning with chain-of-thought (CoT) prompting, achieving significant improvements in reasoning capabilities and enabling it to handle more complex logical inference tasks 20.

Meanwhile, Claude, developed by Anthropic, introduces the concept of Constitutional AI (CAI), emphasizing the helpfulness, harmlessness, and truthfulness of model outputs, making it particularly suitable for sensitive application areas with strict requirements for safety and ethical standards 21. For example, in a study comparing responses to cancer-related patient questions, Claude outperformed physicians in empathy, quality, and readability, highlighting its potential in ethically sensitive medical communication 22. Gemini, developed by Google DeepMind, is characterized by a native multimodal architecture, enabling the coordinated processing of text, images, audio, video, and code within a unified framework, significantly enhancing cross-modal understanding and reasoning capabilities 23. Additionally, the Llama series released by Meta, as the first open-source LLM available for commercial use, offers a high degree of flexibility and customizability, allowing researchers and developers to tailor and optimize the models according to specific needs, thus promoting the widespread adoption and innovative development of AI technologies 11.

## Medical applications of LLMs

**Figure 2:**
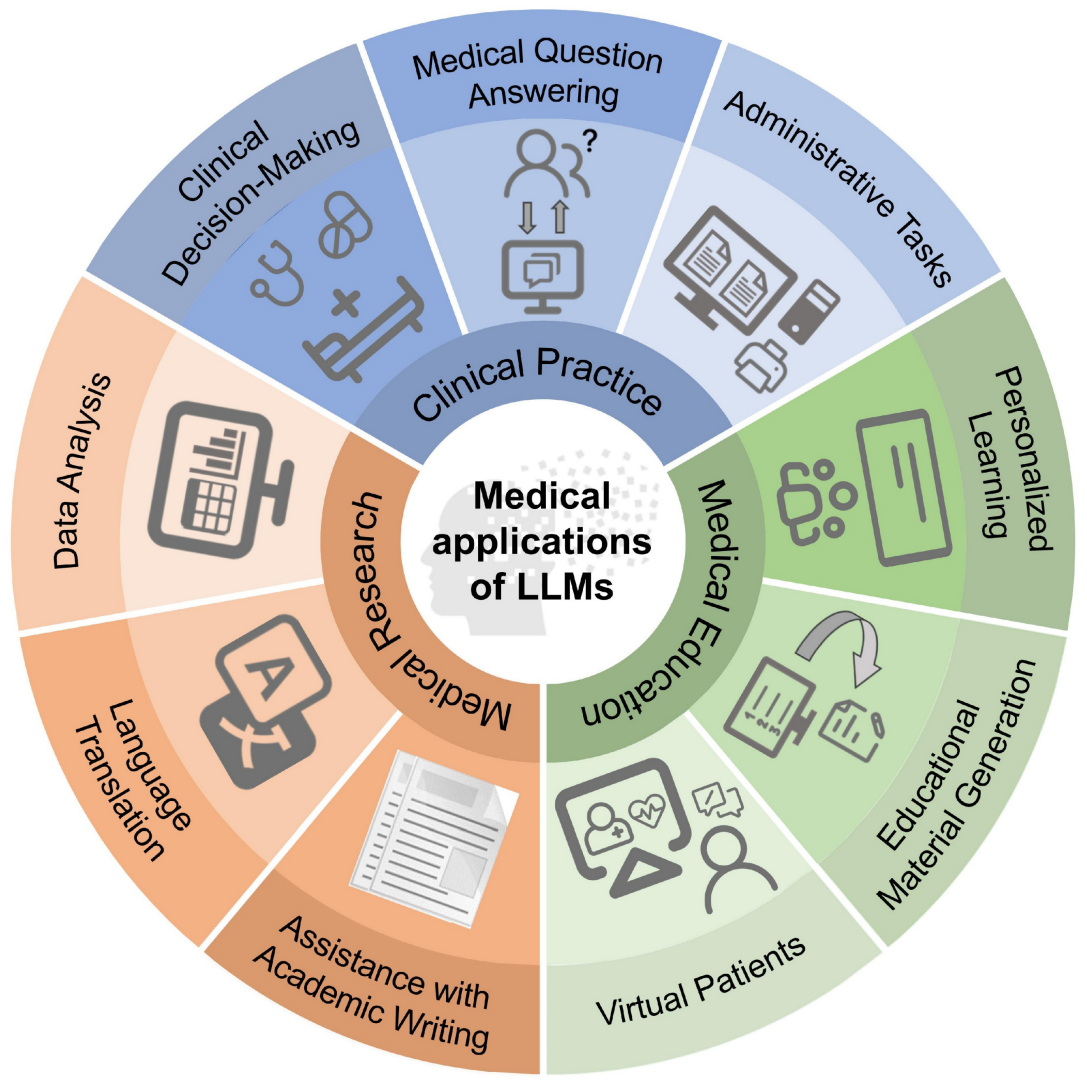
Applications of LLMs in medicine.

Since 2023, LLMs represented by ChatGPT have gradually begun to be applied in the field of medicine, playing an important role in clinical practice, medical research, and medical education (Figure 2).

### Clinical practice

Currently, LLMs have been widely used to assist physicians in clinical decision-making, including initial diagnosis, differential diagnosis, and clinical management. Research showed that based on information such as the history of present illness and physical exam, ChatGPT achieved an accuracy of 60.3% in determining the differential diagnosis. When additional information, such as results of relevant medical tests, was added, ChatGPT's accuracy in narrowing down the final diagnosis increased to 76.9% 24. Notably, LLMs have been shown to surpass the average population consensus in diagnosing rare and complex cases, and they are expected to help address the issues of delayed diagnosis and misdiagnosis in the future 25. Furthermore, recent studies have shown that LLMs play a significant role in aiding decision-making within clinical subspecialties like neurology and cardiology, for example, in diagnosing Alzheimer's disease and managing valvular heart diseases 26, 27.

LLMs have a wide range of applications in the field of medical question answering. They can not only answer a variety of patient questions regarding diagnosis, treatment, and management of diseases 28, but also help interpret the results of laboratory tests 29 and even provide emotional support 30. Furthermore, the application of LLMs can improve the performance of clinical risk prediction based on structured electronic health records 31. For example, a retrospective study found that LLMs with additional pre-training performed excellently in predicting the risk of recurrence after an initial seizure-like episode 32. In patient triage, a cross-sectional study showed that LLMs could accurately assess the criticality of a patient's condition with performance comparable to that of a resident physician 33. Thus, LLMs are promising to be incorporated into emergency department workflows to improve the efficiency and accuracy of emergency triage.

The application of LLMs in the field of radiology similarly shows broad prospects. Studies have shown that assisted generation of radiology reports using LLMs not only improves efficiency and quality but also helps surgeons make more accurate surgical decisions 34, 35. Moreover, LLMs can simplify radiology reports, improving their readability to facilitate patient understanding 36, 37. In clinical work, LLMs have the potential to automate administrative tasks and outperform medical experts in multiple tasks dealing with clinical text 38, 39. Therefore, applying LLMs to the optimization of clinical workflows can effectively reduce the documentation burden on medical staff, enabling them to focus more on patients 40, 41.

### Medical research

With the popularity of LLMs, an increasing number of medical researchers have begun to utilize them to write academic papers. While LLMs can generate seemingly logical and fluent “academic papers” in a short time, such papers are likely to contain factual errors, logical fallacies, and even fabricated references, among other problems 42. This has undoubtedly aroused concerns within the academic community about the authenticity and originality of the papers. On the other hand, LLMs also show the potential to assist scientific research. For example, they can help physicians quickly review a large amount of literature and generate abstracts, and they can also help authors with language translation and polishing 43, thereby improving the efficiency of scientific research. Despite the great potential of LLMs in academic writing, the boundaries of their use remain undefined, and the related ethical issues urgently need to be discussed 44. In addition to article writing, LLMs also demonstrate promising potential for applications in systematic reviews and meta-analyses. For example, as a tool for literature selection, LLMs exhibit high sensitivity and specificity, which can effectively improve work efficiency 45, 46.

LLMs also demonstrate powerful data processing and analysis capabilities in medical research. For example, they can transform unstructured data, such as medical records and test results, into extractable structured data, providing stronger data support for medical research 47. In addition, research showed that compared with traditional statistical software (SAS, SPSS, and R), GPT-4 demonstrated numerous advantages in data analysis, such as more efficient analysis, and a more friendly and intuitive user interface. In the future, LLMs are expected to become a powerful auxiliary tool for statistical analysis and further promote the development of medical research 48.

### Medical education

LLMs have broad application prospects in medical education, covering various aspects such as personalized learning, educational material generation, and student assessment 49. During the learning process, LLMs can serve as virtual teaching assistants, providing personalized guidance and feedback, and timely adjustment of teaching strategies according to students' individual differences and learning progress 50. For example, it can automatically generate more targeted practice questions based on students' answering performance, helping them consolidate their knowledge and address gaps. LLMs can also simulate real patients, conducting interactive dialogues with medical students for training and assessment of clinical skills such as history taking and diagnostic reasoning 51, 52. Similar to standardized patients, they offer realistic and versatile training scenarios for medical students. Studies showed that this personalized learning approach based on LLMs could effectively improve students' learning interest, engagement, and learning outcomes 53. Additionally, LLMs can be used in medical exams, such as automatically generating high-quality multiple-choice questions to reduce the burden on teachers 54, 55. An increasing amount of research indicates that medical students have positive attitudes toward the application of LLMs to assist medical education 56. It is foreseeable that LLMs will continue to drive transformation in medical education and provide new possibilities for training future medical professionals.

## Challenges and Future Development Directions of LLMs in Medicine

This section will explore in depth the challenges faced by LLMs in medicine and propose corresponding strategies for their future development (Table 1).

### Hallucination

Hallucination of LLMs refers to the generation of results that are meaningless or inconsistent with the provided source content 57. In the medicine field, LLMs may generate responses that include fictitious drug recommendations or cite non-existent clinical studies as supporting evidence. Such hallucinations may lead to misdiagnosis, inappropriate treatment, and incorrect medical management. Therefore, it is crucial to reduce the phenomenon of hallucinations to ensure the accuracy and reliability of the output results produced by LLMs.

To address this issue, researchers have proposed several effective strategies, including fine-tuning, reinforcement learning from human feedback (RLHF), and retrieval-augmented generation (RAG). Fine-tuning refers to retraining a pre-trained model on a specific domain dataset, such as medical data, to improve its task adaptability. For example, Clément Christophe et al. applied a combination of instruction-tuning and parameter-efficient tuning to the LLaMA-2 model using a large-scale medical question-answering dataset, which significantly improved the model's accuracy on the USMLE benchmark and effectively reduced the occurrence of hallucinations 58. RLHF leverages human feedback to optimize the model's output behavior, aiming to better align it with human values and expectations. This technique has been widely applied in mainstream LLMs such as ChatGPT and Claude, further reducing the occurrence of hallucinations in medical question answering and complex reasoning tasks 59. RAG is a method that retrieves external knowledge such as clinical guidelines in advance and incorporates it into the generation process to ensure that the output is grounded in factual information. A representative example is the MedGraphRAG framework proposed by Junde Wu et al., which incorporates graph-based medical retrieval to significantly improve model performance on multiple medical benchmark tests 60. Nowadays, RAG is increasingly recognized as a key strategy for mitigating hallucinations in medical LLMs. Furthermore, the combination of LLMs and knowledge graphs (KGs) are considered an effective approach to address the hallucination problem 61. For example, Dawei Li et al. proposed DALK, a dynamic collaborative enhancement framework for LLMs and KGs. Research results based on the Alzheimer's Disease question-answering benchmark show that DALK outperforms other AI technique in overall performance 62.

In the future, addressing hallucination issues will rely on the integration and optimization of techniques such as fine-tuning, RLHF, and RAG. The synergy of multiple strategies is expected to further enhance the accuracy and reliability of model outputs, laying a solid foundation for their widespread application in medicine.

### Interpretability

The interpretability of LLMs refers to their capacity to explain their decision-making process in a manner that is comprehensible to humans and elucidate the relationship between inputs and outputs 63. However, the majority of current LLMs are 'black-box' models with opaque internal workings that make it difficult to explain their predictions 64. This poor interpretability leads to a number of problems. Firstly, healthcare professionals and patients may be unable to comprehend and trust the clinical decisions and medical recommendations generated by the models, which greatly restrict the application of LLM in medicine. Secondly, researchers lack understanding of their internal mechanisms, making it difficult to identify potential flaws in LLMs, thereby limiting the improvement of their performance.

Nowadays, in order to overcome this challenge, multidisciplinary collaboration has become an inevitable trend in the development of medical LLMs. Medical experts should be deeply involved in the model development process, integrating professional medical knowledge into model training. They also need to evaluate and correct the model's outputs to ensure that they conform to medical logic and clinical practice. For example, a recent study proposed a multidisciplinary collaborative framework based on role-playing agents to enhance the medical knowledge comprehension and reasoning ability of LLMs by simulating multiple rounds of medical expert discussions 65. In addition, it has been demonstrated that GPT-4 is capable of simulating the cognitive process of doctors and providing accurate diagnostic outcomes by guiding diagnostic reasoning with specific prompts. This discovery also brings hope for solving the 'black box' problem of LLMs, demonstrating their potential for interpretability in medicine 66.

### Evaluation benchmarks

At present, due to the lack of unified evaluation benchmarks, medical professionals are unable to objectively and comprehensively compare the performance of different LLMs. Therefore, it is difficult to judge the accuracy and reliability of the model's output results, which severely limits the application of LLMs in real clinical scenarios 67.

Recently, researchers have made significant efforts in this regard and proposed a series of evaluation benchmarks for medical LLMs. For example, Singhal, K. et al. proposed MultiMedQA, an evaluation benchmark that is closer to human standards. It covers multiple aspects of professional medicine, medical research, and patient consultation, used to evaluate the model's ability in the medical question-answering 13. BenchHealth, another evaluation benchmark, introduces multidimensional metrics like relevance, fidelity, comprehensiveness, generalizability, and robustness to more comprehensively assess model performance 68. However, existing evaluation benchmarks predominantly focus on closed-ended medical question-answering tasks 69, 70. This assessment approach is difficult to reflect the complexity of real clinical settings, as doctors often need to answer open-ended questions with no predefined options in actual clinical practice, based on the specific circumstances of patients 68. Therefore, future research needs to focus on developing evaluation benchmarks that are more closely aligned with real-world medical scenarios, thus better promoting the standardized application of LLMs in medicine.

### Data limitations

The training and evaluation of LLMs rely on large-scale, diverse, and representative datasets 71. However, in the medical field, accessing, processing, and using clinical data faces several challenges that severely limit the development of medical LLMs. Firstly, access to clinical data is constrained by strict ethical, legal, and privacy protections. Authorization for use can only be granted after a complex approval process, which results in relatively few datasets that are available for training LLMs 72. Secondly, medical data usually needs to be manually annotated by experienced medical experts to ensure its accuracy and professionalism. This process is not only time-consuming and labor-intensive but also poses a significant challenge to data processing efficiency 73. Given that high-quality data is crucial for training and evaluating LLMs, acquiring and processing clinical data efficiently and securely becomes a key prerequisite for promoting the widespread use of LLMs in medicine.

Furthermore, medical diagnosis and treatment often require the integration of multimodal information, such as textual medical histories, and imaging results, for comprehensive judgment 74. Single-modal text data is no longer sufficient to meet the demand for multi-source heterogeneous data analysis in medicine. For this reason, researchers have begun to explore the application of multimodal LLMs in medicine 75. For example, in radiology, multimodal LLMs can combine imaging images and corresponding text reports to assist physicians in making more accurate imaging diagnoses 76. In dentistry, researchers are trying to use multimodal LLMs to integrate patients' oral images and speech symptom descriptions, achieving fully automated diagnosis of oral diseases 77. Med-PaLM M, proposed by Tu T et al., is one of the successful cases of multimodal LLM applications in medicine. It can flexibly encode and interpret multiple types of biomedical data, and shows great potential for application in disease diagnosis, treatment recommendation, and drug development 78.

### Energy consumption

The training and inference processes of LLMs are accompanied by considerable energy consumption and are heavily dependent on high-performance graphics processing units (GPUs), such as NVIDIA's A100 and H100. Studies have shown that executing any meaningful inferences with the 65B LLaMA model requires at least eight V100 GPUs with 32GB of memory each, or four A100 GPUs with 80GB of memory each 79. However, most hospitals and healthcare institutions, particularly those in resource-limited regions, generally lack the infrastructure and financial capacity to support the continuous operation of such energy-intensive AI systems. This presents a significant challenge for the deployment and application of LLMs in the field of medicine.

In recent years, continuous progress in energy-efficient model design has provided feasible directions for addressing this challenge. Techniques such as quantization, knowledge distillation, sparsification, pruning, and mixture-of-experts (MoE) architectures have enabled researchers to significantly reduce the computational demands of LLMs while preserving their performance 80. DeepSeek-R1, for instance, employs a MoE architecture that selectively activates only task-relevant model parameters, thereby reducing computational cost during inference while sustaining strong performance in specialized domains 81, 82. In addition to algorithmic optimizations, emerging low-power hardware technologies, such as memristor crossbar architectures, have shown promising potential for enabling energy-efficient deployment of LLMs 83. Future research should aim to develop medical LLMs that combine high performance with energy efficiency, thereby facilitating their broad and sustainable application in healthcare.

### Ethical concerns

The application of LLMs in medicine faces numerous ethical challenges. 1. Data privacy and security: LLMs require massive amounts of patient data during training. In the absence of comprehensive security measures, the models could potentially memorize and disclose this information during the training process, thus threatening patient privacy and data security 6. 2. Fairness and bias: If the dataset is biased, for example, if there is insufficient data on certain races, genders, or socioeconomic statuses, the model's output results may be biased, leading to unfair distribution of healthcare resources or irrational diagnosis and treatment protocols 84. 3. Liability determination: When LLMs are applied to assist in clinical decision-making, there are currently no consensus for determining liability in cases where the model provides incorrect recommendations that lead to adverse outcomes. 4. Academic integrity: The powerful text generation capabilities of LLMs have been used by some scholars to write medical papers 85, 86 and even to generate false research data and images 87, 88, which raises concerns about academic integrity.

Therefore, it is crucial to give high priority to ethical issues in the development and application of LLMs in medicine. Under the premise of ensuring that everyone can benefit equally from the medical LLMs, we need to actively explore and establish robust ethical guidelines and regulatory mechanisms to protect patient privacy and prevent data misuse. At the same time, rigorous clinical trial validation is required to ensure the safety and efficacy of medical LLMs. Currently, clinical studies on the application of LLMs in the field of medicine are still relatively limited 7. In the future, more prospective clinical trials are needed to evaluate the performance of LLMs in real clinical settings to avoid potential risks.

## Conclusions

The rapid development of LLMs in medicine is exciting, however, the challenges they face are equally significant and cannot be ignored. Improving the accuracy and interpretability of models, addressing the lack of evaluation benchmarks and data, energy consumption and related ethical issues will be the focus of future research. Notably, despite their improving performance on medical tasks, LLMs are currently not capable of replacing human physicians, particularly in complex clinical decision-making. Under appropriate ethical and safety safeguards, rigorously validated LLMs have the potential to become valuable tools for optimizing clinical workflows and improving communication between doctors and patients. Looking ahead, the development of medical LLMs requires the joint participation of medical professionals, AI specialists, ethicists, and experts from other fields. By establishing unified evaluation benchmarks, developing multimodal LLMs, and conducting more prospective clinical trials, LLMs are expected to break through the existing bottlenecks, provide patients with more accurate and personalized healthcare services, and help smart healthcare move to a higher level.

## Figures and Tables

**Table 1:** Summary of Challenges and future development directions of LLMs in medicine.

Challenges	Description	Future development directions
**Hallucination**	Sometimes, the outputs generated by LLMs may appear reasonable, but they actually do not align with the user's input, contradict prior context, or are inconsistent with the facts.	- Fine-tuning: Retraining a pre-trained model on domain-specific data, such as medical texts, to enhance its performance on specialized tasks. - RLHF: Leveraging human feedback to optimize model outputs and better align them with human values and expectations. - RAG: Retrieving external knowledge, such as clinical guidelines, prior to generation to ensure that model outputs are grounded in factual information.
**Interpretability**	It refers to the ability of LLMs to reveal their internal reasoning chains and decision-making processes in a manner that is comprehensible to humans. However, the 'black box' nature of LLMs greatly reduces user trust and reliability of results.	- Guiding diagnostic reasoning through specific prompts: By utilizing specific structured prompts, LLMs are able to correlate the patient's history, symptoms, and ancillary examinations to form a clear chain of reasoning. - Multidisciplinary Collaboration: Medical experts collaborate with AI specialists in the development of LLMs for optimizing their decision-making pathways.
**Evaluation benchmarks**	Current medical LLMs still lack extensive and comprehensive evaluation benchmarks that can reflect real clinical workflows. Therefore, it is difficulty to systematically measure and compare the performance of different LLMs.	- Utilizing desensitized real electronic health records and medical literature to construct more representative and challenging evaluation datasets that better simulate actual clinical environments. - Designing more complex evaluation tasks, such as diagnostic reasoning, treatment recommendation, and doctor-patient dialogue generation, to more comprehensively assess the model's overall capabilities.
**Data limitations**	Due to ethical concerns and the highly specialized nature of the medical field, the acquisition, processing, and use of clinical data are severely restricted, significantly hindering the development of medical LLMs. Single text data is no longer sufficient to meet the needs of medical diagnosis and treatment.	- Establishment of standardized data-sharing models. - Development of new techniques for data annotation and pre-processing to improve the quality and efficiency of data processing. - Multimodal LLMs: These models can simultaneously process and understand medical data in multiple modalities, such as text, images, and speech, to achieve more comprehensive and accurate medical information analysis and knowledge reasoning.
**Energy consumption**	The training and inference of LLMs demand substantial energy and rely on high-performance GPUs. However, many hospitals, especially in resource-limited regions, lack the infrastructure and funding to sustain such energy-intensive AI systems.	- Algorithmic optimization: Techniques such as quantization, knowledge distillation, sparsification, and pruning reduce computational demands. - Hardware innovation: Emerging low-power hardware, such as memristor crossbar architectures, enables more energy-efficient deployment of LLMs.
**Ethical concerns**	Data privacy and security. Fairness and bias. Liability determination. Academic integrity.	- Establishment of robust ethical guidelines and regulatory measures. - Conducting more prospective clinical trials in the future to provide solid scientific evidence for the practical application of LLMs in medicine.
